# Volatile DMNT systemically induces jasmonate-independent direct anti-herbivore defense in leaves of sweet potato (*Ipomoea batatas*) plants

**DOI:** 10.1038/s41598-019-53946-0

**Published:** 2019-11-22

**Authors:** Anja K. Meents, Shi-Peng Chen, Michael Reichelt, Hsueh-Han Lu, Stefan Bartram, Kai-Wun Yeh, Axel Mithöfer

**Affiliations:** 1 Research Group Plant Defense Physiology, Max Planck Institute for Chemical Ecology, 07745 Jena, Germany; 2 Institute of Plant Biology, and Climate Change/Sustainable Development Center, National Taiwan University, Taipei, 106 Taiwan; 3 Department of Biochemistry, Max Planck Institute for Chemical Ecology, 07745 Jena, Germany; 4 Department of Natural Product Biosynthesis, Max Planck Institute for Chemical Ecology, 07745 Jena, Germany; 5 Present Address: Sanming Academy of Agricultural Sciences, Shaxian, Fujian 365000 China

**Keywords:** Chemical biology, Ecology, Plant sciences

## Abstract

Plants perceive and respond to volatile signals in their environment. Herbivore-infested plants release volatile organic compounds (VOCs) which can initiate systemic defense reactions within the plant and contribute to plant-plant communication. Here, for *Ipomoea batatas* (sweet potato) leaves we show that among various herbivory-induced plant volatiles, (*E*)-4,8–dimethyl–1,3,7-nonatriene (DMNT) had the highest abundance of all emitted compounds. This homoterpene was found being sufficient for a volatile-mediated systemic induction of defensive Sporamin protease inhibitor activity in neighboring sweet potato plants. The systemic induction is jasmonate independent and does not need any priming-related challenge. Induced emission and responsiveness to DMNT is restricted to a herbivory-resistant cultivar (Tainong 57), while a susceptible cultivar, Tainong 66, neither emitted amounts comparable to Tainong 57, nor showed reaction to DMNT. This is consistent with the finding that *Spodoptera* larvae feeding on DMNT-exposed cultivars gain significantly less weight on Tainong 57 compared to Tainong 66. Our results indicate a highly specific, single volatile-mediated plant-plant communication in sweet potato.

## Introduction

Plants are constantly subjected to different kinds of biotic stress. Especially herbivorous insects pose as a major threat based on their variability in ways of attacking the plant. An inevitable consequence of herbivory is the mechanical wounding of the infested tissue together with the introduction of signaling compounds from the feeding organisms that can be recognized by the plant to initiate the appropriate defense reactions^1^. Particularly chewing insects from the orders Lepidoptera and Coleoptera can cause severe tissue damage but simultaneously trigger distinct defense-related signaling pathways in the plant^2,3^. Generally, strategies in plant defense are classified as direct or indirect^4^. Direct defense strategies often rely on compounds that are toxic, repellent or anti-nutritive and contribute directly to the plants’ defense^4^. Indirect defense describes the involvement of additional trophic levels apart from the host and the feeding insect. For instance, the production and emission of herbivore-induced plant volatiles (HIPV) such as terpenoids or fatty acid derivatives (green leaf volatiles), can attract predators and/or parasitoids of the herbivores and reduce infestation^5,6^. This generation and emission of HIPVs is regulated by phytohormones, in particular jasmonates^7^. In general, phytohormones play an essential role in regulatory processes during plant defense. Beyond the jasmonates, i.e. jasmonic acid (JA) and its active form jasmonoyl-isoleucine (JA-Ile), salicylic acid (SA), abscisic acid (ABA), and ethylene have been identified as signaling molecules that mediate and orchestrate the defense against pathogen and herbivore attacks^8,9^.

Important for the success of plant defense against herbivores is a coordinated local and systemic communication between cells of the infested tissue and distant organs. As recent studies demonstrated, for that purpose plants own fast, systemic, stress-related signaling mechanisms that include electrical signals, Ca^2+^ ions, and reactive oxygen species (ROS) traveling within the vascular system in order to trigger systemic responses in a plant under attack^10–13^. However, here only the leaves that are connected via the vascular system are integrated. In addition, it was shown that especially HIPVs can also be involved in signaling thereby inducing defense-related genes or priming systemic tissue within the plant as well as in a plant-plant communication where neighboring plants may receive information about an upcoming herbivore threat^14–20^.

Sweet potato (*Ipomoea batatas* Lam.; Convolvulaceae) is one of the most important tuber crops worldwide with a rich phenotypic variability exhibited in many cultivars. Especially the development of insect-resistant cultivars attracted attention in agricultural sciences because sweet potato is subjected to a tremendous variety of pests. *I. batatas* has a high nutritional value, which is mainly due to an abundant storage protein, Sporamin, that is constitutively present in the tuberous roots. This protein also gained importance as a defensive protein against herbivores as it has insect–defense features due to its trypsin protease inhibitory (TPI) activity^21,22^. Previous studies showed that overexpression of *Sporamin* in tobacco or sweet potato led to severe growth retardation in larvae of *Spodoptera litura*, confirming the ability of this sweet potato trypsin inhibitor to confer insect resistance^21^. Sporamin protease inhibitor (SPI) induction in the leaves depends on jasmonates^23,24^. Recently, functional studies in sweet potato showed that the NAC-domain transcription factor, *Ib*NAC1, positively regulates *SPI* expression and thus contributes to the protection against wounding and herbivory^25^.

Strikingly, upon inflicted mechanical damage on sweet potato leaves, *SPI* is locally but even stronger systemically induced in both leaves and stems^22,23^. This finding raised the question for the nature of the systemic signaling and the underlying mechanisms, in particular because *I. batatas* stems can reach a length of several meters; thus, a solely involvement of the vascular system seems unlikely and VOC mediated signaling is conceivable as discussed^19,26^.

The overall goal of our study was to identify signals or compounds that are involved in and responsible for the systemic defense induction after mechanical wounding and herbivory in both an insect herbivory resistant (Tainong 57, TN57) and a susceptible (TN66) cultivar of *I. batatas*. Therefore, we analyzed wounding and herbivory-induced phytohormones including jasmonates, *SPI* expression, its inherent trypsin-inhibitor activity, and emission of volatile compounds in local and systemic leaves as defense-related readouts. In addition, we tested the impact of inducible defense in both cultivars on the performance of the insect herbivore *S. litura*. The knowledge gained from the present study provides better understanding of plant-plant communication in sweet potato. It highlights potential resistance traits that can be targeted in order to develop new approaches, which increase plant resistance against herbivore attacks.

## Results and Discussion

### Induction of defensive *sporamin* and jasmonate accumulation

Previous studies have shown that a complex wounding-inducible signaling cascade is activated by different modes of damage in sweet potato plants^22–25,27^. Strikingly, *SPI* transcripts were found to accumulate preferentially in non-wounded compared to wounded leaves^22^. We decided to first re-examine this finding and performed experiments using herbivorous insect larvae of the generalist lepidopteran species *Spodoptera littoralis* or the robotic caterpillar MecWorm (MecW)^28^ mimicking only the mechanical wounding part during herbivory. After either treatment of the 3rd fully expanded sweet potato TN57 leaf for 0.5, 1 and 3 h, both the treated local leaf and the non-treated adjacent 4th entirely developed systemic leaf, which are not directly connected via the vascular system (Supplementary Fig. S1), were harvested for qRT-PCR to investigate SPI gene expression levels. While *S. littoralis* treatment induced only a small transient increase of *SPI* level in the local leaf, systemically, feeding resulted in a significant increase of *SPI* transcripts after 0.5 h (nearly 6-fold; Supplementary Fig. S2a). SPI gene expression after MecW treatment showed no increase in the local leaf whereas in the systemic leaves a clear trend of *SPI* upregulation (up to 8-fold after 1 h; Supplementary Fig. S2b) was observed. These findings confirm and further support the hypothesis that the wounding-inducible signaling cascade in sweet potato - which ultimately contributes to the plants’ protection against the herbivore by the production of the trypsin inhibitor - is triggered mainly systemically.

**Figure 1:**
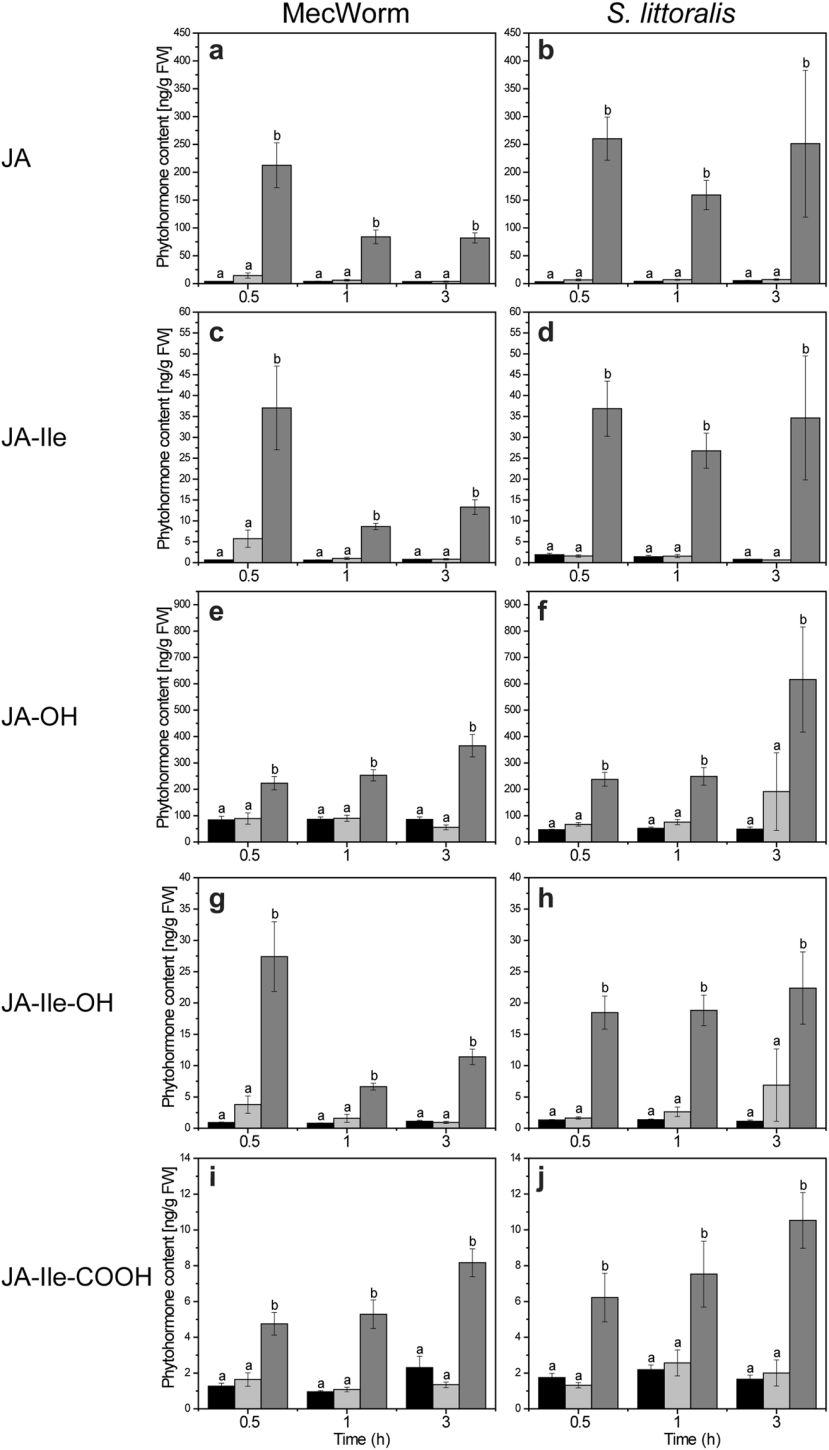
Local but no systemic increase of jasmonates after wounding in *Ipomoea batatas*. (**a**–**j**) Jasmonate levels after mechanical wounding by MecW (**a**,**c**,**e**,**g**,**i**; n = 10–11) and insect feeding by *S. littoralis* (**b**,**d**,**f**,**h**,**j**; n = 7–12) measured in *I. batatas* TN57 after 0.5 h, 1 h and 3 h. Phytohormone levels were measured in locally wounded leaves (dark gray bars) and the adjacent unwounded systemic leaf (light gray bars). Leaves from undamaged plants served as controls (black bars). Statistically significant differences between each treatment group after treatment were analyzed for each time point separately using one–way ANOVA. Different letters indicate significant differences among groups for p < 0.05, determined by Tukey’s test. In (**a**–**j**), data are presented as mean ± SEM.

In order to elucidate further whether the bioactive JA-Ile and other phytohormones play a role in local and systemic defense regulation, MecW-wounded and *S. littoralis*-fed TN57 leaves were analyzed for phytohormone levels. Compared to non-treated controls, the concentrations of both JA and JA-Ile showed a significant increase locally in the MecW-wounded leaves as well as in the *S. littoralis-*treated leaves at all time points (Fig. 1a–d). Strikingly, in contrast to the strong local response throughout both treatments, systemic leaves showed no significant differences compared to control plants (Fig. 1). So far, in sweet potato little was known about the production of other jasmonates apart from JA. Here, in addition to JA, JA-Ile was demonstrated to accumulate after both types of treatment: mechanical wounding and herbivory (Fig. 1c,d). Moreover, a pronounced accumulation of jasmonate metabolites was found; i.e. after the rapidly enhanced production of JA and JA-Ile in wounded and infested leaves, metabolites such as JA-OH, JA-Ile-OH and JA-Ile-COOH were detected as well. The accumulations of these metabolites occurred in a similar manner as found for JA and JA-Ile (Fig. 1e–j). This confirms the catabolism of JA and JA-Ile, which has been described by Wasternack and Hause^9^.

Upon mechanical wounding, SA only showed a slight but significantly elevated level in the damaged leaf (MecW) after 3 h of treatment (Supplementary Fig. S3a). Analyses of the drought–stress related phytohormone abscisic acid (ABA) showed no obvious differences to control plants (Supplementary Fig. S3b). Also, SA levels were not significantly affected by herbivore feeding (Supplementary Fig. S3c) but herbivory treatment generated a significant amount of ABA in the damaged leaf compared to the untreated control after 3 h of treatment (Supplementary Fig. S3d). In conclusion, there was no induced production of stress–related phytohormones in local leaves that was nearly comparable to what was found for jasmonates.

Since there was no detectable accumulation of jasmonates after herbivore or MecW treatment in unwounded sweet potato leaves but an induction of *SPI*, systemic activation of the defense-signaling cascade appears to be independent of increased JA levels on-site. These findings contradict previously proposed models by Rajendran *et al*.^24^ and support the possibility of JA-independent pathways for *SPI* induction in unwounded leaf tissues.

Alternatively to a direct jasmonate-dependent upregulation of defense-related proteins, the activation by bioactive hydroxyproline-rich glycopeptides (HypSys peptides) was reported by Chen *et al*.^29^. These cell wall localized peptides are processed from wound- and jasmonate- inducible sweet potato precursor proteins with the ability to induce expression of *SPI* in *I. batatas* leaves. Thus, it is conceivable that the activation of *SPI* expression in unwounded sweet potato leaves is triggered without JA accumulation in the target leaves but by endogenous *Ib*HypSys peptides. However, an initial herbivory- or wounding-induced JA burst in the local, treated leaf seems necessary to start the whole cascade of signals and those peptides mainly act locally. Nevertheless, an example of JA-independent systemic defense responses has been observed in *Arabidopsis* plants in which the herbivore-induced accumulation of *γ*-amino-butyric acid (GABA) follows the same pattern^30^. Overall, these considerations lead to the conclusion that an accumulation of phytohormones in systemic tissues is not essential; at least for a systemic induction of defense-related genes like *SPI*.

### Upregulated volatile emission in response to wounding and herbivory

Excluding the involvement of jasmonates in systemic defense upregulation in sweet potato TN57, other putative signaling mechanisms were investigated. Although the mechanism of volatile perception in plants remains so far unknown^4^, VOCs can have a systemic effect on neighboring or within plants. These compounds may serve as a direct elicitor (e.g. methyl jasmonate), as a precursor for further transformation into a defensive compound^31^ or by priming the adjacent plants in preparation for an imminent herbivore infestation^20,32^. In order to test whether VOCs released by wounded sweet potato plants can activate defense mechanisms in neighboring plants, a first proof-of-principle experiment was conducted. Two *I. batatas* plants were placed in a closed glass tank (34 L) without any physical contact; one plant was mechanically wounded using tweezers^24,25,27^ with the second plant remaining non-wounded. After 24 h, in the non-wounded plants gene expression of *SPI* and the upstream transcription factor *IbNAC1* were analyzed. Compared to the control, both gene transcripts were found to be significantly induced (Supplementary Fig. S4).

**Figure 2:**
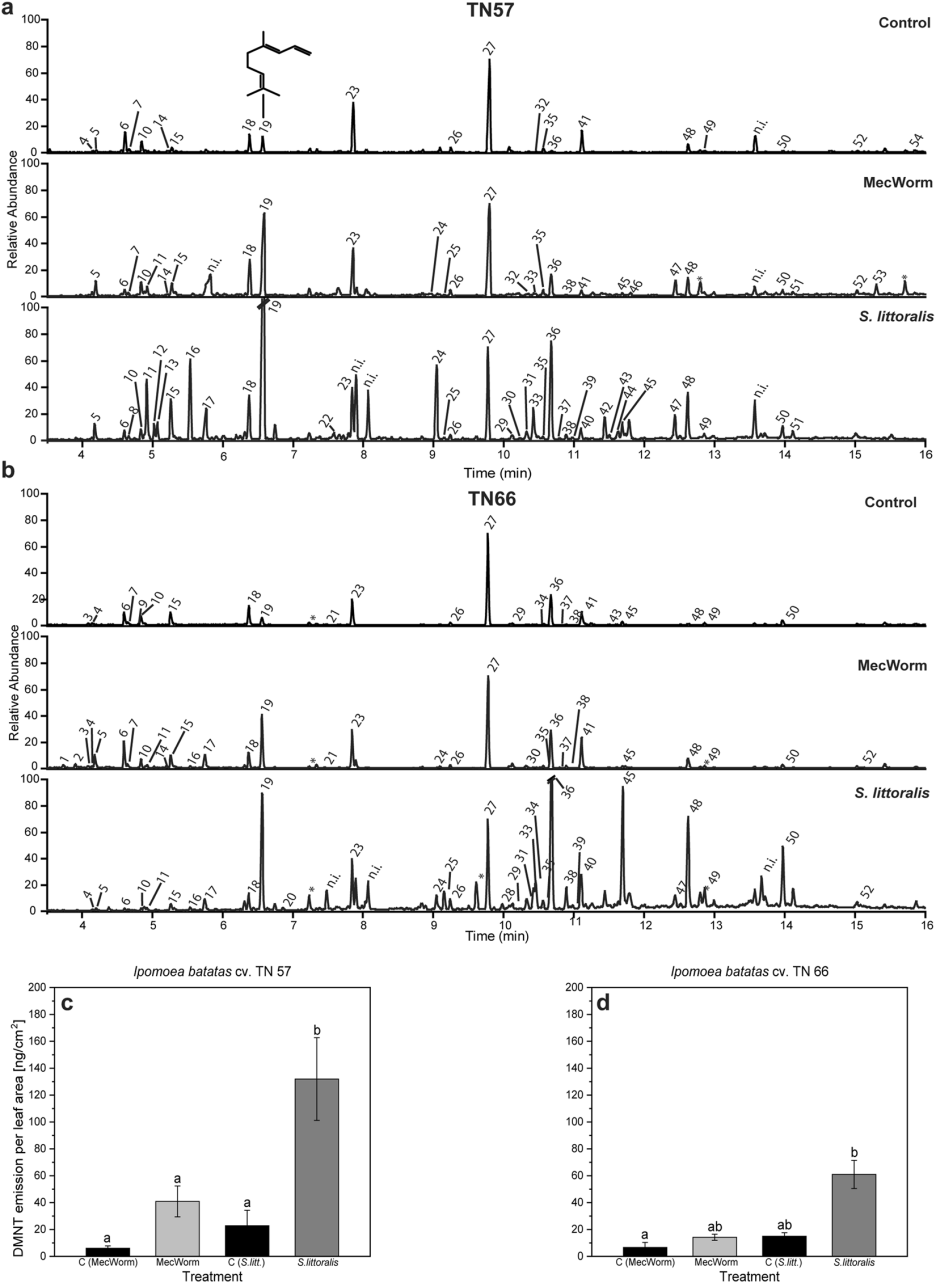
Volatile emission and upregulation of (*E*)-4,8–dimethyl–nonatriene (DMNT) in *Ipomoea batatas*. (**a**,**b**) Gas chromatograms of volatiles emitted by *I. batatas* TN57 (**a**) and TN66 (**b**): Controls without wounding; volatiles induced by mechanical damage (MecW) inflicted over 18 h; volatiles induced by feeding of *S. littoralis*. All volatiles were collected over 24 h and eluted with internal standard. Asterisks mark contamination by plasticizer or column residuals. Identified compounds are marked as follows: (1) *α*-Pinene; (2) 1-Butoxy-2-propanol; (3) 2-Ethylhexanal; (4) Benzaldehyde; (5) 5-Ethyl-(5 H)-furan-2-one; (6) 6-Methyl-5-hepten-2-one; (7) Mesitylene; (8) 1-Decene; (9) *n*-Decane; (10) *n*-Octanal; (11) *(Z)-*Hex-3-enyl acetate; (12) Hexyl acetate; (13) *(E)-*Hex-2-enyl acetate; (14) Limonene; (15) 2-Ethyl-hexanol; (16) *(E)-*β-Ocimene; (17) unidentified monoterpenoid (93, 136); (18) *n*-Nonanal; (19) 4,8-Dimethylnona-1,3,7-triene; (20) Phenyl acetonitrile; (21) Naphthalene; (22) *(Z)-*Hex-3-enyl butanoate; (23) *n*-Decanal; (24) Indole; (25) *n*-Tridecane; (26) *n*-Undecanal; (27) Internal standard (*n*-bromodecane); (28) *(E)-*2-Undecenal; (29) α-Copaene; (30) β-Cubebene; (31) 7-epi-Sesquithujene; (32) 1-Tetradecene; (33) *(Z)-*Jasmone; (34) *n*-Tetradecane; (35) Dodecanal; (36) *(E)-*β-Caryophyllene; (37) β-Copaene; (38) *(E)-*α-Bergamotene; (39) Sesquisabinene; (40) α-Humulene; (41) Geranyl acetone; (42) Germacrene D; (43) β-Ionone; (44) Bicyclogermacrene; (45) *n*-Pentadecane; (46) Tridecanal; (47) Nerolidol; (48) (3E,7E)-4,8,12-Trimethyltrideca-1,3,7,11-tetraene; (49) *n*-Hexadecane; (50) *n*-Heptadecane; (51) *n*-Pentadecanal; (52) *n*-Octadecane; (53) Isopropyl tetradecanoate; (54) *n*-Hexadecanol. Identification of compounds is shown in Supplementary Table S1. (**c**,**d**) DMNT emission after mechanical damage and herbivore feeding in *I. batatas* TN57 (**c**) and TN66. (**d**) VOCs were collected over 24 h with 18 h mechanical wounding by MecW (light gray bars; n = 5–8) or 24 h infestation with *S. littoralis* (dark gray bars; n = 7–12) and the respective control (black bars; n = 5–7). Bars represent the mean ± SEM of emitted DMNT in ng cm^−2^ leaf area. Statistically significant differences between each group were analyzed using a Kruskal-Wallis one-way ANOVA on ranks. Different letters indicate significant differences among groups for p < 0.05, determined by Dunn’s test and adjusted p-values according to Benjamini & Hochberg. (**c**) TN57: p < 0.001. (**d**) TN66: p = 0.004.

**Figure 3:**
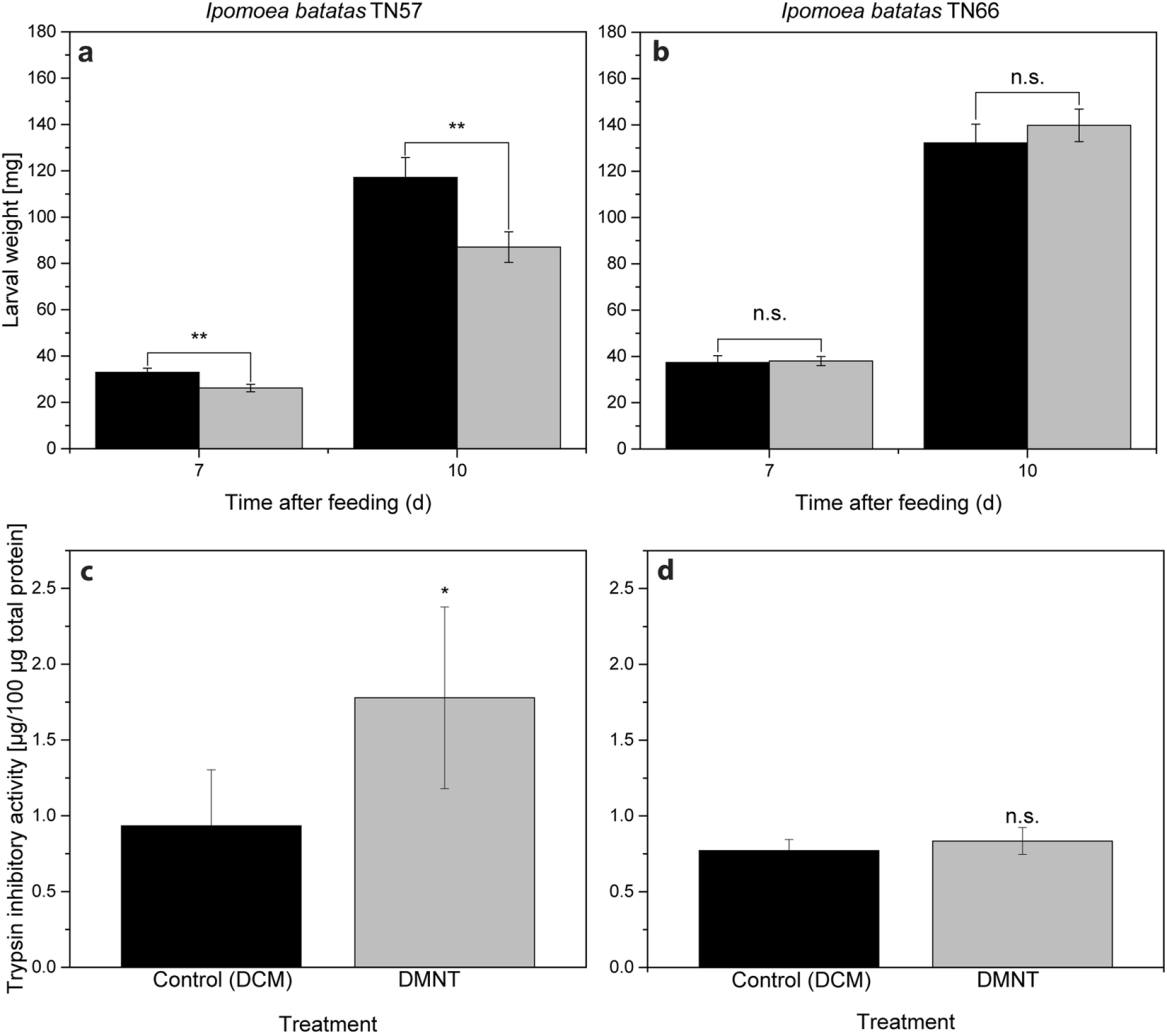
Airborne DMNT increases defense capabilities in *Ipomoea batatas* TN57. (**a**,**b**) Larval weight of *S. litura* after feeding for 7 d and 10 d on DCM (control) or DMNT- treated TN57 (**a**, n = 16) or TN66 (**b**, n = 25) plants. For DMNT treatment, plants were incubated with 3.9 nM for 3 h. (**c**,**d**) Trypsin inhibitory activity of TN57 (**c**, n = 5) and TN66 (**d**, n = 6) after incubation with 3.9 nM of DMNT for 3 h. Bars represent the mean ± SEM of larval weight or trypsin inhibitory activity for control (DCM, black bars) and DMNT treatment (gray bars). Significance levels are indicated by the asterisks (n.s. = non-significant; *p < 0.05; **p < 0.01). (**a**) TN57: p (CxDMNT) = 0.002; (**b**) TN66: p (CxDMNT) = 0.468. (**c**) TN57: p (CxDMNT) = 0.028; (**d**) TN66: p (CxDMNT) = 0.207. Asterisks indicate significant differences between control and DMNT treatment, based on a t-test (**c**,**d**) and ANOVA followed by a Tukey- adjusted comparison based on a linear model (**a**,**b**).

For a deeper analysis, volatile compounds released by *I. batatas* after biotic/abiotic damage were collected and identified. Application of mechanical damage (MecW) and herbivore feeding (*S. littoralis*) on single sweet potato leaves led to an emission of at least 40 identified compounds (Supplementary Table S1) in TN57 with differences in terms of volatile bouquet quality as well as in quantities of individual compounds (Fig. 2a and Supplementary Table S1)^28,33^. Investigation of emitted volatiles in unwounded control plants of *I. batatas* TN57 and a second cultivar TN66 showed a basic composition comprising different classes of hydrocarbons including alkenes, aldehydes and mono-, sesqui-, and homoterpenes. Focusing on TN57, mechanical damage for 18 h revealed an emission of additional compounds such as (*Z*)-hex-3-enyl acetate, (*Z*)-jasmone or nerolidol. Apart from the previously detected compounds, feeding of *S. littoralis* resulted in a more complex volatile blend (Fig. 2a and Supplementary Table S1). Multiple sesquiterpenes like α-copaene, α-humulene, germacrene D as well as another monoterpene, (*E*)-β-ocimene, were added to the volatile bouquet. The homoterpenes (*E*)-4,8-dimethyl-1,3,7-nonatriene (DMNT) and (*E,E*)-4,8,12-trimethyl-1,3,7,11-tridecatetraene (TMTT) as well as (*E*)-β-caryophyllene and also indole and pentadecane increased upon *Spodoptera* treatment. Compared to all other compounds, a strikingly enhanced emission of DMNT was observed during treatments (Supplementary Table S2). Thus, the amount of emitted DMNT per cm^2^ leaf area after both types of wounding was determined in TN57 (Fig. 2c). Control values showed that the experimental set-ups already triggered the emission of small amounts of DMNT due to the fixation of the leaf in a plexiglas cabinet (MecW) or enclosing the leaf into a feeding cage with additional bagging into plastic foil (*S. littoralis* feeding). The latter procedure – being more invasive compared to the MecW treatment - resulted in an even higher amount of emitted DMNT in the *S. littoralis* control samples. Nevertheless, feeding of *S. littoralis* resulted in a significantly higher emission of DMNT in comparison with MecW wounding and controls.Damaging the leaf with MecW for 18 h resulted in an 6-fold increased averaged total emission after 24 h of volatile collection compared to the control. DMNT emission during 24 h was even more pronounced in herbivore–infested leaves resulting in an average amount of 132 ng ± 31 DMNT cm^−2^ leaf area. Differences between emitted DMNT upon insect feeding and other treatments were shown to be significantly different (Fig. 2c). These results suggested that the strong increase of DMNT emission might play a role in the interaction of sweet potato against herbivores. Thus, in addition to TN57, we analyzed the volatile emission of the susceptible sweet potato TN66 using the same treatments (Fig. 2b; Supplementary Tables S1 and S2). In general, the VOC patterns in the non-treated control and after MecW treatment are only slightly different compared to the patterns found for TN57. Unwounded TN66 control plants emitted 25 detectable compounds with an even higher number of VOCs during MecW treatment (32 VOCs). The variety of volatiles emitted after mechanical wounding (30 VOCs) or control (22 VOCs) in TN57 was slightly lower. Upon *S. littoralis* feeding less compounds showed up in TN66 (33 identified VOCs) than in TN57 with 38 detectable emitted VOCs (Fig. 2; Supplementary Table S1). Regarding DMNT, after both types of treatment the emission of this particular homoterpene was also less pronounced in TN66 (Fig. 2d). The exact quantification of DMNT released from TN66 showed that neither after MecW nor *S. littoralis* treatment a significant increase was detectable. Again, DMNT emission upon larval feeding was significantly higher than upon sole mechanical wounding. The increased number of the individual compounds released after herbivore infestation can be explained by the combination of mechanical wounding with the contribution of HAMPs (herbivory-associated molecular patterns) provided by oral secretion^1^. In TN57, indole was induced only after herbivory while among all VOCs, DMNT showed the most pronounced upregulation after both MecW and herbivory, followed by caryophyllene. The very strong induction of DMNT upon both treatments was the main reason to choose this compound for further experiments. The most prominent compounds after feeding in TN66 were DMNT, TMTT, (*E*)-β-caryophyllene and pentadecane. Previous studies also showed the variability of emitted compounds by *I. batatas* in a cultivar-specific manner^34,35^ and the resulting effect on the behavior of specialist herbivores^36^. The latter study focused mainly on root-emitted volatiles; we focus on emitted volatiles from leaves and their putative protective effect against feeding generalists like *Spodoptera* (Fig. 3). Because the emission of DMNT is a common phenomenon after insect feeding, though in a species- and herbivore- dependent manner^28,37,38^, especially DMNT with its many biological functions^39,40^ might be a regulator in wound-inducible signaling within and among sweet potato plants.

### Airborne DMNT increases herbivore resistance in *I. batatas* TN57

To explore further, whether airborne DMNT alone is sufficient to induce defense responses and enhanced herbivore resistance in undamaged *I. batatas*, we synthesized DMNT and incubated this VOC (at comparable amounts that were emitted from treated leaves, i.e. app. 6 µg leaf^−1^) together with sweet potato plants in a closed glass tank (34 L). Either DMNT or dichloromethane (DCM, serving as solvent for DMNT dilutions) as the respective control were applied on a cotton ball to avoid any physical contact with the plant and was incubated in adjusted concentrations and durations. The induction of defense-related SPI gene expression was fast; the incubation with 20 µg DMNT in a volume of 34 L (3.9 nM) showed already after 15 min an 11-fold upregulation of *SPI* (Supplementary Fig. S5a). This concentration of DMNT applied for 1 h resulted in an even higher *SPI* upregulation (21-fold) in sweet potato leaves while lower concentrations, i.e. 5 µg in the same volume, showed no detectable induction (Supplementary Fig. S5b), suggesting a concentration dependent reaction.

**Figure 4:**
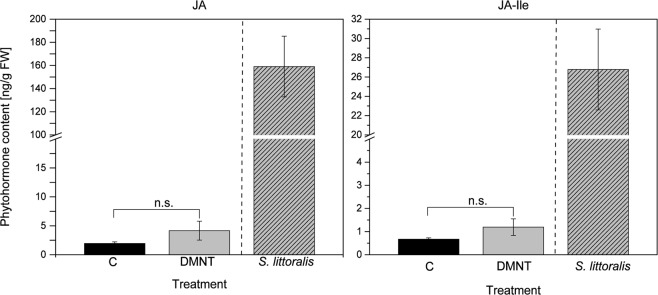
DMNT does not systemically induce jasmonate production. JA and JA-Ile levels of single *I. batatas* TN57 plants incubated with 1.41 µg of DMNT dissolved in dichloromethane in 2.4 L glass desiccators for 1 h (light gray bar, n = 10). Black bars indicate the control samples treated with dichloromethane (n = 7). *S. littoralis* feeding (light gray with stripes) was used as a positive control for the visualization of jasmonate induction. Bars represent the mean ± SEM of detected JA and JA-Ile. Significant differences were determined using a Shapiro–Wilk normality test and a subsequent Mann-Whitney rank sum test for the treatment and the respective control (n.s = non-significant); p JA (CxDMNT) = 0.130; p JA-Ile (CxDMNT) = 0.661.

Based on these findings, further experiments were performed to elucidate whether DMNT-treated plants show increased resistance against herbivores. Therefore, sweet potato plants were exposed for 3 h to 3.9 nM DMNT and subsequently treated with *S. litura* for several days. By exposing *S. litura* larvae to volatile DMNT it was demonstrated beforehand that DMNT itself had no toxic effect on the larvae (Supplementary Fig. S6). A significantly reduced larval weight compared to the control treatment could already be observed after feeding for 7 d on TN57. This effect became even more pronounced after 10 d of feeding (Fig. 3a). In contrast to TN57, in TN66 no differences in the larval performance after feeding either on DMNT-exposed or control plants could be detected (Fig. 3b). After determining the *SPI* transcript accumulation, the trypsin inhibitory activity was analysed. Compared to the control treatment, DMNT-exposed TN57 plants displayed a significantly higher trypsin inhibitory activity, whereas in TN66 no induced activity was detected (Fig. 3c,d). Regarding the finding that herbivory and wounding induced jasmonates only locally, the ability of DMNT to induce JA or JA-Ile itself was investigated in the systemic leaves. As shown in Fig. 4 and consistent with the former results, no significant induction of jasmonates was measured.

Thus, we could demonstrate that the external application of synthetic DMNT to both sweet potato cultivars caused a significant induction of TPI activity only in TN57, which likely results in a reduced larval performance when feeding on those TN57 leaves.

In contrast, TN66 was more susceptible to insect feeding due to the lack of TPI-depending defense upregulation (Fig. 3). Based on these findings TN57 appears to be not only more effective in DMNT release but also more sensitive to DMNT compared with TN66, resulting in the induction of defense mechanisms.

Overall, DMNT was identified as a potent trigger for cultivar-specific systemic defense upregulation. Whether or not other VOCs such as caryophyllene or indole may have a similar function needs to be investigated in further studies.

Cultivar-specific volatile emission of sweet potato is known to play a crucial role in attraction of specialist attackers^35,36^. However, here not the plants’ defense but their attractiveness towards the herbivore (*Cylas formicarius*) made them more susceptible or resistant. Herbivory-induced volatile release is also involved in the communication within and between plants. HIPV can be perceived and prime the receiving tissues and plants for a rapid and enhanced response upon subsequent wounding and insect attack^41^. It is well known that some green leaf volatiles (GLV) have priming activities in various plant species such as maize, Arabidopsis, tomato, and wheat^16,42–44^. Moreover, for maize it was recently shown that pretreatment with indole can increase the production of JA-Ile, ABA and certain volatiles after wounding, representing another example for priming^17^. For lima bean (* Phaseolus lunatus)* it has been shown that volatile blends emitted by *Tetranychus urticae*-infested lima bean plants as well as single compounds such as GLV but also DMNT were able to induce expression of some defense-related genes in neighboring plants^14,15^. Beyond priming or gene induction, the upregulation of a distinct direct defensive activity in neighboring plants simply by a volatile signal has never been shown for a crop plant. To our knowledge, the only example for a direct defense is β-ocimene emitted from *Myzus persicae*-infested *Brassica pekinensis* plants that was recently shown to induce glucosinolates in un-infested plants^45^. Strikingly, our study not only confirmed that in sweet potato upregulation of defense by VOCs alone is possible; it showed also that this was independent of jasmonate accumulation (Fig. 4). In addition, in TN57 for an increased TPI activity induction no further treatment was necessary, which does not represent a classical priming scenario.

**Figure 5:**
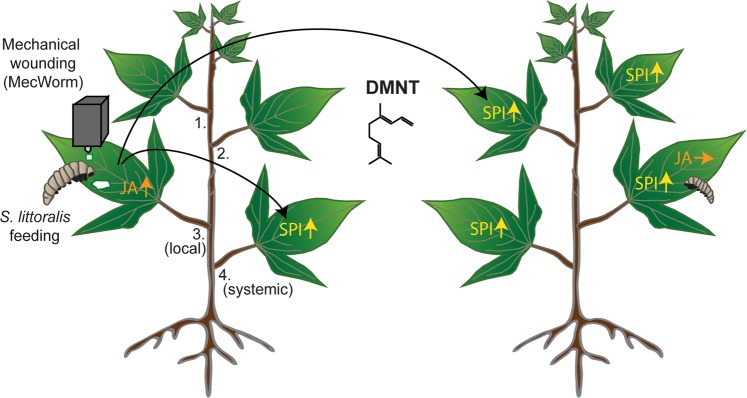
Model of DMNT emission that triggers SPI-dependent resistance enhancement in TN57 after mechanical wounding and *Spodoptera* herbivory. Upon mechanical wounding (MecWorm) or herbivore (*S. littoralis)* feeding, jasmonates (JA) are locally upregulated in the treated leaf. Sporamin protease inhibitor (SPI) is upregulated mainly systemically. In parallel, (*E*)-4,8–dimethyl–nonatriene (DMNT) is emitted to the environment and induces the generation of SPI in leaves of non-treated neighboring *I. batata*s plants without changes in JA levels. As a consequence, these plants show higher resistance against feeding *Spodoptera* larvae.

All of these findings show a cultivar-dependent specificity of volatile-mediated defense in sweet potato with DMNT as a signal compound able to trigger protective mechanisms for resistance against herbivores in non-attacked conspecific TN57 plants (Fig. 5). Next, it is necessary to study how DMNT and other VOCs function on the molecular level as has been shown for mint volatiles in soybean where histone acetylation in the promotor regions of defense genes caused enhanced RNA levels^46^. The ability to emit DMNT conjoined with the corresponding volatile perception ability emphasizes the relevance of this signaling compound for the defense in cultivar TN57. Morphological distant but adjacent parts of the same plant might also interact via volatiles, thereby omitting long-distance signaling along the vascular connections within the plant. Such a volatile shortcut may represent an efficient protection mechanism to enhance plant resistance against attackers. However, in particular systemic signaling within the plant may rely on a combination of both the vascular system and volatiles^26^.

In any case, the demonstrated DMNT-SPI relation in sweet potato can serve as a promising model for plant-plant communication. Nevertheless, in the near future it is essential to gain more information on the systemic signaling processes in *I. batatas* during herbivory to further improve the resistance against insect pests. Identification and generation of cultivars with higher natural emission levels of DMNT might be useful to strengthen the overall resistance of this crop in the field.

## Materials and Methods

### Plant material and growth conditions

Sweet potato scions (*Ipomoea batatas* Lam.; cultivars Tainong 57 and Tainong 66) were grown in a substrate mix (200 L Klasmann TS1 mixed with 70 L Klasmann Tonsubstrat, Klasmann- Deilmann, Germany) in 10 cm diameter round pots under long day conditions (16 h light:8 h dark) at 28 °C (day) and 25 °C (night) and 70% relative humidity for 3 weeks. With light, illumination in the growth chamber was kept at 100 µmol m^−2^ s^−1^. All plants were fertilized once per week with 0.01% Ferty Basisdünger 3 (Planta Düngemittel, Germany). Plants used for studies at NTU (National Taiwan University, Taipei) were cultivated under the same growth conditions at a temperature of 25 °C (day) and 20 °C (night). Experiments conducted at MPICE in Jena were set up between 9 and 12 o’clock (am). In each experiment, 3-week-old plants with six to eight fully expanded leaves were used. Each 3rd fully expanded leaf was locally treated (MecWorm or *S. littoralis* feeding) and harvested together with the adjacent 4th leaf (systemic) using scissors.

### Mechanical wounding with MecWorm

In order to mimic herbivore feeding without its elicitors in the oral secretion, MecWorm^28^, known as the mechanical caterpillar was used to inflict continuous mechanical wounding to the 3rd fully developed leaf of each plant. Wounding sites of rectangular shapes were set with a punching speed of 12 punches per min lasting for 30, 60 and 180 min. For headspace volatile collection the continuous damage was extended to 18 h. Additionally, the treated leaf was enclosed in a Plexiglas cabinet of the MecWorm apparatus.

### Insect rearing

*Spodoptera littoralis* (Boisd., Lepidoptera, Noctuidae) larvae were hatched from eggs (Bayer Cropscience, Germany) and reared on an artificial diet consisting of 500 g hackled beans, 9 g ascorbic acid, 9 g 4-ethylbenzoic acid, 9 g vitamin E Mazola oil mixture (7.1%), 4 ml formaldehyde, 1.2 l water, 1 g-sitosterol, 1 g leucine, 10 g AIN-76 vitamin mixture and 200 ml agar-water solution (7.5%). Insects were reared at 23–25 °C with a photoperiod of 14 h. Experiments conducted at the National Taiwan University applied 2nd-instar *Spodoptera litura* larvae for insect feeding assays. *S. litura* larvae were provided by Prof. Y. Wu (Department of Entomology, NTU). Twenty-four hours prior to herbivore application, 3rd to 4th–instar larvae of *S. littoralis* or 2nd–instar larvae of *S. litura* were separated into plastic cups for starving.

### Herbivore infestation with *S. littoralis* larvae

For herbivore treatment with *S. littoralis*, the third fully expanded leaf of each plant was infested with a single 3rd to 4th–instar larva of *S. littoralis* and enclosed in a feeding cage for 0.5, 1 or 3 h. Modifications for volatile collection included that the treated leaf and the feeding herbivore with the surrounding feeding cage were enclosed in odorless PET foil (Bratschlauch, Toppits, Germany) for 24 h. All herbivore experiments were conducted in the growth chamber. Measuring of the treatment period started after the first observed feeding by the herbivore.

### RNA extraction and quantitative real time (qRT)-PCR

Collected sweet potato leaves were ground to a fine powder in liquid nitrogen. RNA was extracted using TRIzol Reagent (Invitrogen, USA) by adding 1 ml reagent to 100 mg of ground leaf tissue. After thorough mixing, all samples were incubated at room temperature for 10 min. The following centrifugation steps were performed at 12,000 × g at 4 °C. After incubation, samples were centrifuged for 15 min and the supernatant was transferred into 200 µl chloroform. Subsequently samples were inverted for 1 min and centrifuged again for 15 min. Addition of 200 µl chloroform to the supernatant was repeated and samples were centrifuged for 15 min. The aqueous phase was added to approximately 600 µl isopropanol (1:1, sample: isopropanol) and precipitated overnight at 20 °C. For sedimentation, samples were centrifuged for 15 min and the supernatant was removed. RNA pellet was washed with 1 ml of cooled 80% ethanol, mixed and centrifuged for 15 min. The washing step was repeated once more and the resulting pellet was vacuum-dried in an Eppendorf Concentrator plus (Eppendorf AG, Germany) for 10 min at 30 °C. Subsequently, the dried pellet was dissolved in 50 µl of preheated water for 15 min at 42 °C with mixing every 5 min. RNA concentration was measured using NanoDrop One microvolume UV-Vis spectrophotometer (Thermo Fisher Scientific, USA). First strand cDNA was synthesized from 6 µg of total RNA using RevertAid First Strand cDNA synthesis Kit (Thermo Fisher Scientific, USA) with Oligo(dT)_18_ Primer. Synthesis was conducted according to the manufacturers’ instructions with slight modifications. After mixing 6 µg of template RNA with primer and water, additional incubation at 65 °C for 5 min with subsequent chilling on ice for 10 min was conducted before adding the reaction mix. The sample was incubated at 42 °C for 60 min. The reaction was stopped at 72 °C for 5 min. Subsequently cDNA was diluted by addition of 40 µl of water for a total cDNA concentration of 100 ng µl^−1^.

Real-time qPCR analysis was performed using Brilliant II SYBR Green QPCR Master Mix (Agilent Technologies, USA) and gene-specific primers (Supplementary Table S3; Eurofins Genomics, Luxembourg). For normalization of gene expression levels, *IbACTIN-2* was used as housekeeping gene. The master mix was prepared according to the manufacturers’ instructions using 400 nM of each gene-specific primer and 100 ng cDNA per well for a total reaction volume of 25 µl.

Gene amplification was achieved using Bio-Rad CFX96 Real-Time PCR Detection System (Bio-Rad Laboratories, USA) comprising the following steps: 95 °C (3 min); 45 cycles of [95 °C (30 s), 60 °C (30 s), 72 °C (30 s)]; 95 °C (10 s), 65 °C (5 s) and 95 °C (50 s). The generated data was processed by using Bio-Rad CFX Manager (Bio-Rad Laboratories GmbH, USA). For analysis, the normalized fold expression was calculated according to the ΔΔCP method described by Pfaffl^47^. Expression levels were calculated according to the respective control treatment including the gene of interest and the housekeeping gene. For each experiment at least 6 (up to 11) biological replicates were used. Detected CT-values from technical replicates deviating more than 0.5 from each other were not used for calculation. The total RNA from DMNT-induced samples was obtained using the method of Chang, *et al*.^48^. Subsequent cDNA synthesis and qPCR was performed as previously described by Chen, *et al*.^25^.

### Phytohormone extraction and quantification

Collected sweet potato leaves were ground to a fine powder in liquid nitrogen and 200–250 mg of finely ground leaf material was weighed into an Eppendorf tube (Eppendorf AG, Germany). The extraction and detection was performed as previously described by Vadassery, *et al*.^49^ with minor modifications. For phytohormone extraction, weighed powdery leaf material was mixed with 1.5 ml methanol containing 60 ng D_6_–abscisic acid (Santa Cruz Biotechnology, USA), 60 ng of D_6_–jasmonic acid (HPC Standards GmbH, Germany), 60 ng D_4_–salicylic acid (Sigma-Aldrich, USA) and 12 ng of jasmonic acid-^13^C_6_-isoleucine conjugate as internal standard. After brief mixing, samples shook for 30 min at 4 °C using a Rotator Mixer RM-Multi 1 (STARLAB GmbH, Germany) using the program 100 rpm: 15 s, 75 °C: 16 s, 3 °C: 5 s. Samples were centrifuged afterwards at 13,000 × g at 4 °C for 20 min, the supernatant was collected and the remaining pellet resuspended in 500 µl methanol, shaken and centrifuged again as previously described. The combined supernatants were concentrated for 3 h using Eppendorf Concentrator plus (Eppendorf AG, Germany), re-suspended in 500 µl methanol and centrifuged at 16,000 × g at 4 °C for 10 min. Finally, 400 µl of supernatant were used for LC-MS/MS measurements.

Phytohormone analysis was performed using Agilent 1200 HPLC system (Agilent, USA) with subsequent API 5000 tandem mass spectrometer (Applied Biosystems, USA) with a Turbo spray ion source in negative ionization mode. The elution profile was: 0–0.5 min, 10% B; 0.5–4.0 min, 10–90% B; 4.0–4.02 min, 90–100% B; 4.02–4.5 min, 100% B and 4.41–7.0 min, 10% B at a flow rate of 1.1 ml min^−1^.

Multiple reaction monitoring (MRM) was applied to monitor analyte parent ion → product ion: *m/z* 209.1 → 59.0 collision energy (CE) −24 V, declustering potential (DP) −35 V) for jasmonic acid; *m/z* 215.1 → 56.0 (CE −24, DP –35 V) for D_6_-jasmonic acid, *m/z* 322.2 → 130.0 (CE −30 V, DP –50 V) for jasmonic acid-isoleucine conjugate, *m/z* 328.2 → 136.1 (CE −30 V, DP –50 V) for jasmonic acid-^13^C_6_-isoleucine conjugate, *m/z* 136.9 → 93.0 (CE −22 V, DP −35 V) for salicylic acid, *m/z* 140.9 →97.0 (CE −22 V, DP −35 V) for D_4_-salicylic acid, *m/z* 263.0 → 153.2 (CE −22 V, DP −35 V) for abscisic acid, *m/z* 269.0 → 159.2 (CE −22 V, DP –35 V) for D_6_-abscisic acid and *m/z* 290.9 → 165.1 (CE −24 V, DP –45 V) for *cis*-(+)-12-oxophytodienoic acid (*cis*–OPDA).

### Visualization of the plant vascular system

To determine the vascular connections of the sweet potato cultivar used in this study, the stem of 3-week-old *I. batatas* was cut at the plant base and split in two halves. The stem halves were submerged in tap water and commercial purple colored ink (Violett Pelikan 4001, Germany). After 3 h the ink was resorbed and translocated into the leaves of the plant as described in Zimmermann, *et al*.^50^. The staining of the vascular system was monitored with a digital camera.

### Volatile collection and GC–MS analysis

Volatiles were collected over 24 h using the closed-loop stripping technique as previously described by Kunert, *et al*.^51^. Plants were either treated with MecWorm or *S. littoralis* as described above. Each single third and adjacent neighboring fourth leaf, still connected to the whole sweet potato plant, was enclosed in odorless PET foil bags (Toppits, Germany) to avoid contamination by soil volatiles. Each third fully developed leaf of the untreated plant was enclosed in odorless PET bags and used as control. Emission patterns of Plexiglas feeding cages used in herbivore bioassays showed no detectable influence on the induction of volatile release. For constant air circulation and continuous volatile collection each foil bag was connected to a circulation pump (Fürgut GmbH, Germany) attached to a charcoal trap with 1.5 mg absorption material (CLSA filter, 6 cm long, 0.5 cm diameter, Gränicher & Quartero, France). After collection, volatiles were eluted with 2 × 20 µl dichloromethane containing 50 µg ml^−1^ *n*-bromodecane as internal standard. Analysis was conducted using GC-MS (Finnigan TRACE GC 2000, Thermo Fisher Scientific, USA) equipped with a Zebron ZB-5 column (25 m × 0.25 mm × 0.25 µm, Phenomenex, USA) and the following temperature profile for separation of VOCs: Initial temperature was set at 45 °C for 2 min, heating up 10 °C min^−1^ to 200 °C followed by 30 °C min^−1^ to 280 °C. Helium was used as carrier gas with a flow rate of 1.5 ml min^−1^. Split ratio was set at 1: 10 and 1 µl of the eluate was automatically injected. Injector temperature was set to 220 °C. The MS was run in EI mode (70 eV) with a scan range of 35 to 450 amu, a transfer line temperature of 280 °C, and an ion source temperature of 250 °C. Data acquisition was performed using Xcalibur 1.1 (Thermo Fisher Scientific).

### Identification of VOCs

A mixture of n-alkanes C_8_ – C_20_ in *n*-hexane (Sigma-Aldrich, USA) was measured before and after a sample sequence under the same conditions. Retention times of the *n*- alkanes were used to calculate the retention index (RI) for each peak in the GC-MS chromatogram according to the method of van Den Dool and Kratz^52^.

Compounds were tentatively identified based on their mass spectra (MS) in combination with their individual RIs in comparison to Mass Spectral Library (NIST/EPA/NIH)^53^, Adams^54^ and Massfinder^55^ MS and RI databases using Massfinder software in combination with Mass Spectral Library (NIST/EPA/NIH)^53^ MS Search. Authentic reference compounds were used additionally for identification, if at hand. Retention indices deviating more than ±2 from the authentic references and ±5 compared to the database were regarded as mismatches and not considered. For relative quantification, identified peaks of the GC-MS total ion chromatogram (TIC) were integrated and the peak areas were divided by the peak area of the internal standard. According to Massfinder^55^ instructions, peak areas below a minimum peak width of 2 with a sensitivity of 2 and an area threshold of 1000000 were regarded as below the quantification limit. For detailed information, see Supplementary Tables S1 and S2.

### Absolute quantification of DMNT

The absolute amount of emitted DMNT was calculated using a standard curve. DMNT was synthesized as described in Maurer, *et al*.^56^. Different quantities of DMNT were dissolved in pure dichloromethane to generate solutions with the following concentrations of DMNT: 2.5; 5; 10; 20; 50; 100; 150; 200 and 250 µg ml^−1^. All dilutions contained 50 µg ml^−1^ of *n*-bromodecane as internal standard for comparison with the previous volatile measurements. Emitted quantities of DMNT were calculated by division of DMNT peak areas through the respective peak areas of the internal standard. The output value was then inserted into the regression line formula of the measured DMNT standard curve and calculated according to the applied 40 µl elution volume per single leaf and its respective leaf area.

### Induction of plants by volatiles and DMNT

To test whether VOCs of a wounded sweet potato plant can activate defense mechanisms in unwounded neighboring plants, two *I. batatas* plants were placed in a closed glass tank (34 L) without additional air flow and without any physical contact. One plant was then mechanically wounded using tweezers and placed next to an unwounded neighboring plant. Two unwounded plants were placed next to each other in the same type of glass container as control treatment. After 24 h, a single leaf of the non-wounded plants was harvested, used for RNA extraction and subsequent qRT-PCR. Gene expression of *SPI* and the upstream transcription factor *IbNAC1* were then analyzed.

In order to verify induction of defense mechanisms in sweet potato plants by DMNT only and whether there is a concentration- dependency, 5 and 20 µg of DMNT dissolved in 1 ml of pure dichloromethane were impregnated into a single piece of cotton wool to ensure accurate application. The cotton wool was placed without any physical contact centrally between sweet potato plants in a closed glass container (34 L; no additional air flow) for 1 h. As control equal volumes of pure dichloromethane were incubated on cotton wool and placed centrally between three plants.

### Trypsin inhibitory activity assay in sweet potato

After a 3 h- exposure to 3.9 nM DMNT in DCM or pure DCM as control in an enclosed container, the third fully expanded leaf of each treated plant was harvested and used for protein extraction. The leaf material was grinded in liquid N to a fine powder and further homogenized in 2 ml extraction buffer (1x PBS pH 7.4; supplemented with 1 mM PMSF; Santa Cruz Biotechnology, USA). To determine the extracted amount of protein, Bradford assay was performed using Quick Start Bradford 1x Dye Reagent (Bio-Rad, Germany) according to the manufacturer’s instructions. Subsequently, 100 µg of extracted protein were incubated with 2 µg trypsin (Sigma-Aldrich, USA) at 37 °C for 30 min. Afterwards 5 µl N-α-Benzoyl-DL-arginine 4-nitroanilide hydrochloride (50 mg ml^−1^, Sigma-Aldrich, USA) were added and incubated for 20 min with following absorbance measurements at 410 nm using Infinite M200 Microplate reader (Tecan, Switzerland). A regression curve was made using soybean trypsin inhibitor (0, 0.1, 0.2, 0.5, 1 and 2 µg; Sigma-Aldrich, USA) to normalize the quantity of trypsin inhibitor in the total amount of protein.

### Feeding assay

To ensure an authentic volatile exposure, 3-week-old sweet potato plants were incubated with DMNT (20 µl of 1 mg ml^−1^ DMNT in DCM on a cotton ball) or 20 µl pure DCM (control) without any direct physical contact in a glass container (34 L) at 25 °C for 3 h. Afterwards, eight *S. litura* larvae (2^nd^ instar) were placed on the pre-exposed sweet potato plants and allowed to feed for up to 10 d. In order to provide freshly DMNT- induced plants for feeding, the sweet potato plants were replaced after 3 d, 5 d, and 7 d. The larval weights were determined at 7 d and 10 d of feeding using an analytical balance.

### DMNT toxicity assay

Second instar *S. litura* larvae reared on artificial diet were exposed to DMNT (20 µl of 1 mg ml^−1^ DMNT in DCM) or 20 µl pure DCM (control) without direct physical contact in a glass container (34 L) at 25 °C. The larval weights were determined after 10 d of feeding using an analytical balance.

### Statistical analysis

Statistical significances of phytohormone contents were tested using Shapiro–Wilk normality test with a subsequent one–way ANOVA or Kruskal-Wallis one-way ANOVA on ranks in SigmaPlot (V 12.1.0). ANOVA was conducted for each single time point during all treatments. Tukey’s test was selected for all pairwise multiple comparison procedures. Statistical significance between groups was given when p < 0.05. Statistically significant differences of quantified DMNT within each cultivar comparing all treatments were calculated using a Kruskal-Wallis one-way ANOVA on ranks in R (V 3.6.1; R Core Team) followed by Dunn’s test with a Benjamini & Hochberg correction using the package “FSA v0.8.25” (R Core Team). Statistical significance between groups was given when p < 0.05. Relative quantification of emitted volatile organic compounds after mechanical wounding and herbivore feeding were analyzed using SigmaPlot (V 12.1.0). One-sample t-test (against 0) was used for cultivars that only showed a quantifiable amount in a single treatment group whereas samples with two comparable groups were analyzed using Mann-Whitney Rank Sum test. Cultivars emitting a detectable amount in more than two treatments were analyzed with a Kruskal-Wallis ANOVA on ranks. A linear model (lm) was used to examine the effect of DMNT on the larval weight of *S. littoralis*. In this model, the larval weight of *S. littoralis* was set as the dependent variable with treatment and time as the independent variables including the interaction of time and treatment. Significant interactions between the main effects in this model were analyzed using ANOVA. Tukey- adjusted pairwise comparisons of the DMNT treatment with the control based on the model were performed using the package “lsmeans” (R Core Team). These analyses were conducted using R (V 3.6.1; R Core Team). Levels of statistical significance are marked as the following: p < 0.05 (*); p < 0.01 (**) and p < 0.001 (***). Data generated using qRT-PCR was analyzed as described above (see “RNA extraction and quantitative real time (qRT)-PCR”) according to Pfaffl^47^ followed by a Shapiro-Wilk normality test and a one-sample t-test. Statistics for experiments comprising the determination of toxicity and trypsin inhibitory activity were done using Shapiro-Wilk normality test for data exploration followed by a t-test in SigmaPlot (V 12.1.0).

## Supplementary information

Supplementary information

Supplementary Table S1

Supplementary Table S2

## Data Availability

The datasets generated and analysed during the current study are available from the corresponding author on reasonable request.

## References

[1] Mithöfer A, Boland W (2008). Recognition of herbivory-associated molecular patterns. Plant Physiol..

[2] Diezel C, von Dahl CC, Gaquerel E, Baldwin IT (2009). Different lepidopteran elicitors account for cross-talk in herbivory-induced phytohormone signaling. Plant Physiol..

[3] Howe GA, Jander G (2008). Plant immunity to insect herbivores. Annu. Rev. Plant Biol..

[4] Mithöfer A, Boland W (2012). Plant defense against herbivores: chemical aspects. Annu. Rev. Plant Biol..

[5] Kessler A, Baldwin IT (2002). Plant responses to insect herbivory: the emerging molecular analysis. Annu. Rev. Plant Biol..

[6] Takabayashi J, Dicke M (1996). Plant—carnivore mutualism through herbivore-induced carnivore attractants. Trends Plant Sci..

[7] Maffei ME, Mithöfer A, Boland W (2007). Insects feeding on plants: Rapid signals and responses preceding the induction of phytochemical release. Phytochem..

[8] Bonaventure G (2012). Perception of insect feeding by plants. Plant Biol..

[9] Wasternack C, Hause B (2013). Jasmonates: biosynthesis, perception, signal transduction and action in plant stress response, growth and development. An update to the 2007 review in Annals of Botany. Ann. Bot..

[10] Zimmermann MR, Maischak H, Mithöfer A, Boland W, Felle HH (2009). System potentials, a novel electrical long-distance apoplastic signal in plants, induced by wounding. Plant Physiol..

[11] Mousavi SAR, Chauvin A, Pascaud F, Kellenberger S, Farmer EE (2013). Glutamate Receptor-Like genes mediate leaf-to-leaf wound signalling. Nature.

[12] Kiep V (2015). Systemic cytosolic Ca^2+^ elevation is activated upon wounding and herbivory in Arabidopsis. New Phytol..

[13] Choi W-G (2017). Orchestrating rapid long-distance signaling in plants with Ca^2+^, ROS and electrical signals. Plant J..

[14] Arimura G-I (2000). Gene responses in bean leaves induced by herbivory and by herbivore-induced volatiles. Biochem. Biophys. Res. Commun..

[15] Arimura G-I (2000). Herbivory-induced volatiles elicit defence genes in lima bean leaves. Nature.

[16] Engelberth J, Alborn HT, Schmelz EA, Tumlinson JH (2004). Airborne signals prime plants against insect herbivore attack. Proc. Natl. Acad. Sci. USA.

[17] Erb M (2015). Indole is an essential herbivore-induced volatile priming signal in maize. Nat. Commun..

[18] Heil M, Karban R (2010). Explaining evolution of plant communication by airborne signals. Trends Ecol. Evol..

[19] Frost CJ (2007). Within-plant signalling via volatiles overcomes vascular constraints on systemic signalling and primes responses against herbivores. Ecol. Lett..

[20] Frost CJ, Mescher MC, Carlson JE, De Moraes CM (2008). Plant defense priming against herbivores: Getting ready for a different battle. Plant Physiol..

[21] Yeh KW (1997). Sweet potato (*Ipomoea batatas*) trypsin inhibitors expressed in transgenic tobacco plants confer resistance against *Spodoptera litura*. Plant Cell Rep..

[22] Yeh K-W, Chen J-C, Lin M-I, Chen Y-M, Lin C-Y (1997). Functional activity of sporamin from sweet potato (*Ipomoea batatas* Lam.): a tuber storage protein with trypsin inhibitory activity. Plant Mol. Biol..

[23] Wang S-J, Lan Y-C, Chen S-F, Chen Y-M, Yeh K-W (2002). Wound-response regulation of the sweet potato *sporamin* gene promoter region. Plant Mol. Biol..

[24] Rajendran SenthilKumar, Lin I-Winnie, Chen Mei-Ju, Chen Chien-Yu, Yeh Kai-Wun (2014). Differential activation of sporamin expression in response to abiotic mechanical wounding and biotic herbivore attack in the sweet potato. BMC Plant Biology.

[25] Chen S-P (2016). Sweet potato NAC transcription factor, *Ib*NAC1, upregulates sporamin gene expression by binding the SWRE motif against mechanical wounding and herbivore attack. Plant J..

[26] Heil M, Ton J (2008). Long-distance signalling in plant defence. Trends Plant Sci..

[27] Chen S-P, Kuo C-H, Lu H-H, Lo H-S, Yeh K-W (2016). The sweet potato NAC-domain transcription factor IbNAC1 is dynamically coordinated by the activator IbbHLH3 and the repressor IbbHLH4 to reprogram the defense mechanism against wounding. PLoS Genet..

[28] Mithöfer A, Wanner G, Boland W (2005). Effects of feeding *Spodoptera littoralis* on lima bean leaves. II. Continuous mechanical wounding resembling insect feeding is sufficient to elicit herbivory-related volatile emission. Plant Physiol..

[29] Chen Y-C, Siems WF, Pearce G, Ryan CA (2008). Six peptide Wound signals derived from a single precursor protein in *Ipomoea batatas* leaves activate the expression of the defense gene *sporamin*. J. Biol. Chem..

[30] Scholz SS, Reichelt M, Mekonnen DW, Ludewig F, Mithöfer A (2015). Insect herbivory-elicited GABA accumulation in plants is a wound-induced, direct, systemic, and jasmonate-independent defense response. Front. Plant. Sci..

[31] Sugimoto K (2014). Intake and transformation to a glycoside of (Z)-3-hexenol-3-hexenol from infested neighbors reveals a mode of plant odor reception and defense. Proc. Natl. Acad. Sci..

[32] Kessler A, Halitschke R, Diezel C, Baldwin IT (2006). Priming of plant defense responses in nature by airborne signaling between *Artemisia tridentata* and *Nicotiana attenuata*. Oecologia.

[33] Bricchi I (2010). Robotic mechanical wounding (MecWorm) versus herbivore-induced responses: Early signaling and volatile emission in lima bean (*Phaseolus lunatus L*.). Planta.

[34] Horvat RJ, Arrendale RF, Dull GG, Chapman GW, Kays SJ (1991). Volatile constituents and sugars of three diverse cultivars of sweet potatoes [Ipomoea batatas (L.) Lam.]. J. Food Sci..

[35] Korada RR (2013). Plant volatile organic compounds as chemical markers to identify resistance in sweet potato against weevil *Cylas formicarius*. Curr. Sci..

[36] Starr, C. K., Seversonx, R. F. & Kays, S. J. 12. Volatile chemicals from sweet potato and other Ipomoea: Effects on the behavior of *Cylas formicarius*. *Sweet potato Pest Management: A Global Perspective* **235** (1991).

[37] Arimura G-I (2002). Herbivore-induced volatiles induce the emission of ethylene in neighboring lima bean plants. Plant J..

[38] Leitner M, Boland W, Mithöfer A (2005). Direct and indirect defences induced by piercing-sucking and chewing herbivores in *Medicago truncatula*. New Phytol..

[39] Dicke M, Gols R, Ludeking D, Posthumus MA (1999). Jasmonic acid and herbivory differentially induce carnivore-attracting plant volatiles in lima bean plants. J. Chem. Ecol..

[40] Tholl D, Sohrabi R, Huh J-H, Lee S (2011). The biochemistry of homoterpenes - Common constituents of floral and herbivore-induced plant volatile bouquets. Phytochem..

[41] Baldwin IT, Halitschke R, Paschold A, von Dahl CC, Preston CA (2006). Volatile signaling in plant-plant interactions: “talking trees” in the genomics era. Science.

[42] Bate NJ, Rothstein SJ (1998). C6-volatiles derived from the lipoxygenase pathway induce a subset of defense-related genes. Plant J..

[43] Farag MA, Pare PW (2002). C6-Green leaf volatiles trigger local and systemic VOC emissions in tomato. Phytochem..

[44] Ameye M (2015). Priming of wheat with the green leaf volatile *Z*-3-hexenyl acetate enhances defense against *Fusarium graminearum* but boosts deoxynivalenol production. Plant Physiol..

[45] Kang Z-W (2018). Volatile β-ocimene can regulate developmental performance of peach aphid *Myzus persicae* through activation of defense responses in chinese cabbage *Brassica pekinensis*. Front. Plant. Sci..

[46] Sukegawa S, Shiojiri K, Higami T, Suzuki S, Arimura GI (2018). Pest management using mint volatiles to elicit resistance in soy: mechanism and application potential. Plant J..

[47] Pfaffl MW (2001). A new mathematical model for relative quantification in real-time RT-PCR. Nucleic Acids Res..

[48] Chang S, Puryear J, Cairney J (1993). A simple and efficient method for isolating RNA from pine trees. Plant Mol. Biol. Report.

[49] Vadassery J (2012). CML42-mediated calcium signaling coordinates responses to Spodoptera herbivory and abiotic stresses in Arabidopsis. Plant Physiol..

[50] Zimmermann MR, Mithöfer A, Will T, Felle HH, Furch ACU (2016). Herbivore-Triggered Electrophysiological Reactions: Candidates for Systemic Signals in Higher Plants and the Challenge of Their Identification. Plant Physiol..

[51] Kunert, M., David, A., Becher, J. & Boland, W. Volatile sampling from biological sources by the closed-loop-stripping technique. *Cold Spring Harb. Protoc*. **2009**, pdb.prot5233 (2009). 10.1101/pdb.prot5233 20147191

[52] van Den Dool H, Kratz P (1963). A generalization of the retention index system including linear temperature programmed gas—liquid partition chromatography. J. Chromatogr. A.

[53] Mass Spectral Library (NIST/EPA/NIH) (National Institute of Standards and Technology, Gaithersburg, 2014).

[54] Adams, R. P. *Identification of essential oil components by gas chromatography/mass spectrometry*. 4th edn, (Allured, 2007).

[55] Massfinder v. 4.21 (Hochmuth Scientific Consulting, Hamburg, Germany, 2010).

[56] Maurer B, Hauser A, Froidevaux JC (1986). (E)-4,8-Dimethyl-1,3,7-nonatriene and (E,E)-4,8,12-trimethyl-1,3,7,11-tridecatetraene, two unusual hydrocarbons from cardamom oil. Tetrahedron Lett..

