# Supplementary material for: Volatile DMNT systemically induces jasmonate-independent direct anti-herbivore defense in leaves of sweet potato (Ipomoea batatas) plants
Source: Sci Rep. 2019 Nov 22;9:17431. doi: 10.1038/s41598-019-53946-0 (PMC6874613; doi:10.1038/s41598-019-53946-0)
Supplement: Supplementary file 1 — Supplementary information [file 41598_2019_53946_MOESM1_ESM.pdf]

Supplementary Information

Volatile DMNT systemically induces jasmonate-independent direct anti-herbivore defense in leaves of sweet potato (*Ipomoea batatas*)

Anja K. Meents, Shi-Peng Chen, Michael Reichelt, Hsueh-Han Lu, Stefan Bartram, Kai-Wun Yeh, Axel Mithöfer

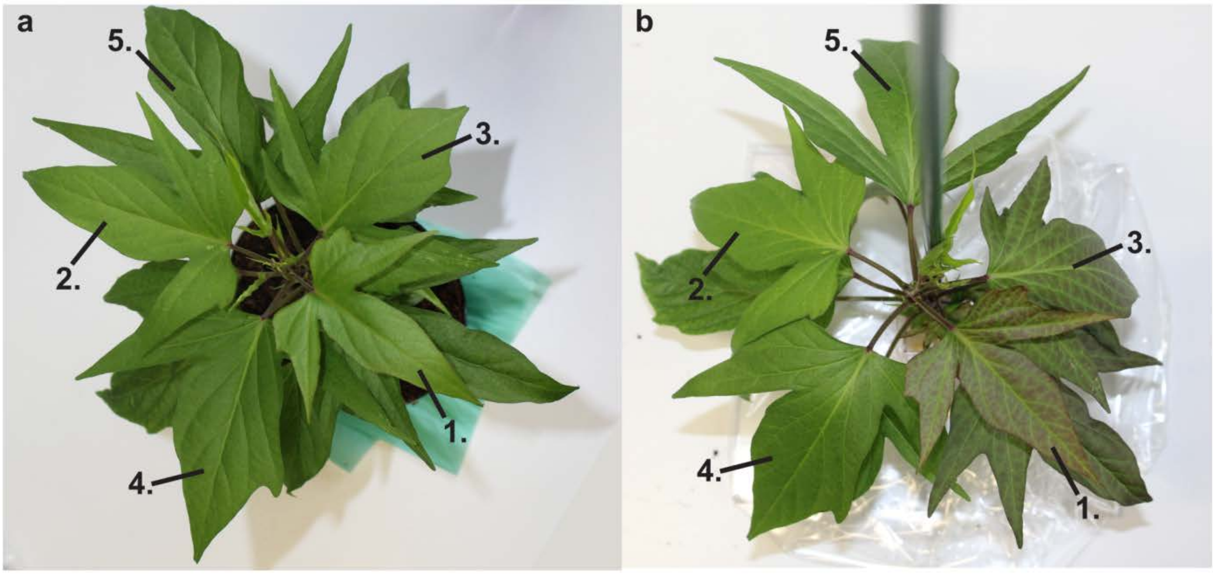

Supplementary Figure S1. No direct vascular connection between adjacent leaves 3 and 4 in 3-week-old *Ipomoea batatas*.

To demonstrate the vascular branching in *I. batatas*, the stems of 3-week-old sweet potato plants were cut at the plant base and split in two halves. The left stem half was placed in tap water with the right stem half submerged in purple ink and incubated for 3 h. (a) *I. batatas* before cutting (t0). (b) Stained *I. batatas* plant after 3 h incubation.

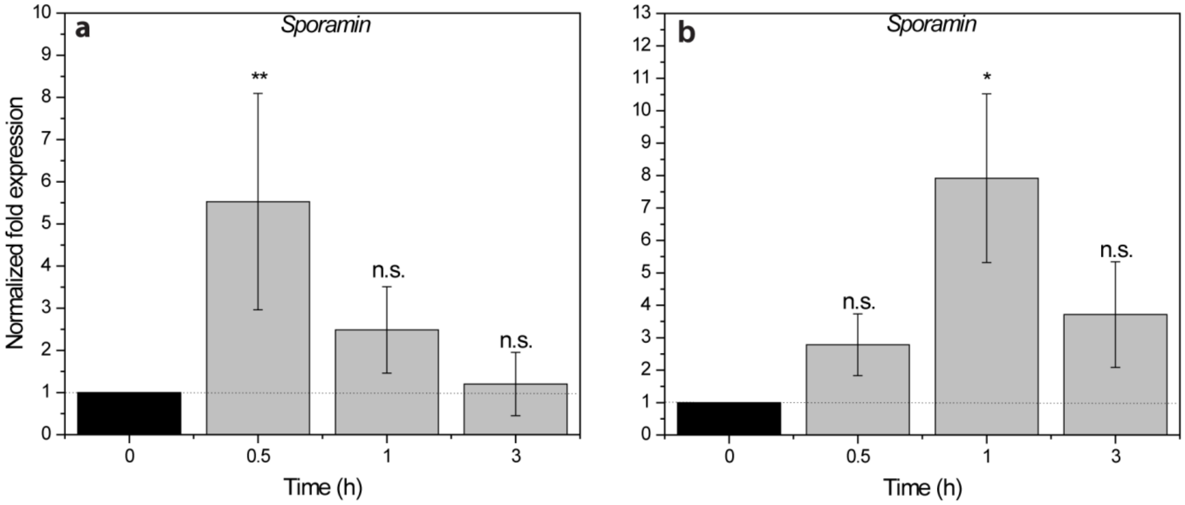

Supplementary Figure S2. Systemic induction of defensive *Sporamin* after *S. littoralis* feeding and MecWorm wounding in *Ipomoea batatas.*

Changes in the transcript level of *Sporamin* were determined in the intact systemic leaves (light gray bars) of *I. batatas* TN57 after 0.5, 1 or 3 h infestation with (a) a single larva of *S. littoralis* (0.5 h: n=10; 1 h: n=11; 3 h: n=6; p (controlx*S. littoralis* 0.5h) = 0.006) or wounding with (b) MecWorm (0.5 h: n=11; 1 h: n=11; 3 h: n=8; p (controlxMecW 1h) = 0.011) relative to the respective untreated control plants (black bar). Subsequent to a Shapiro-Wilk normality test, a one-sample t-test was selected to calculate statistical significant differences within each timepoint. Bars represent the mean ± standard error of the mean (SEM). Significance levels are indicated by the asterisks (*p<0.05;** p<0.01).

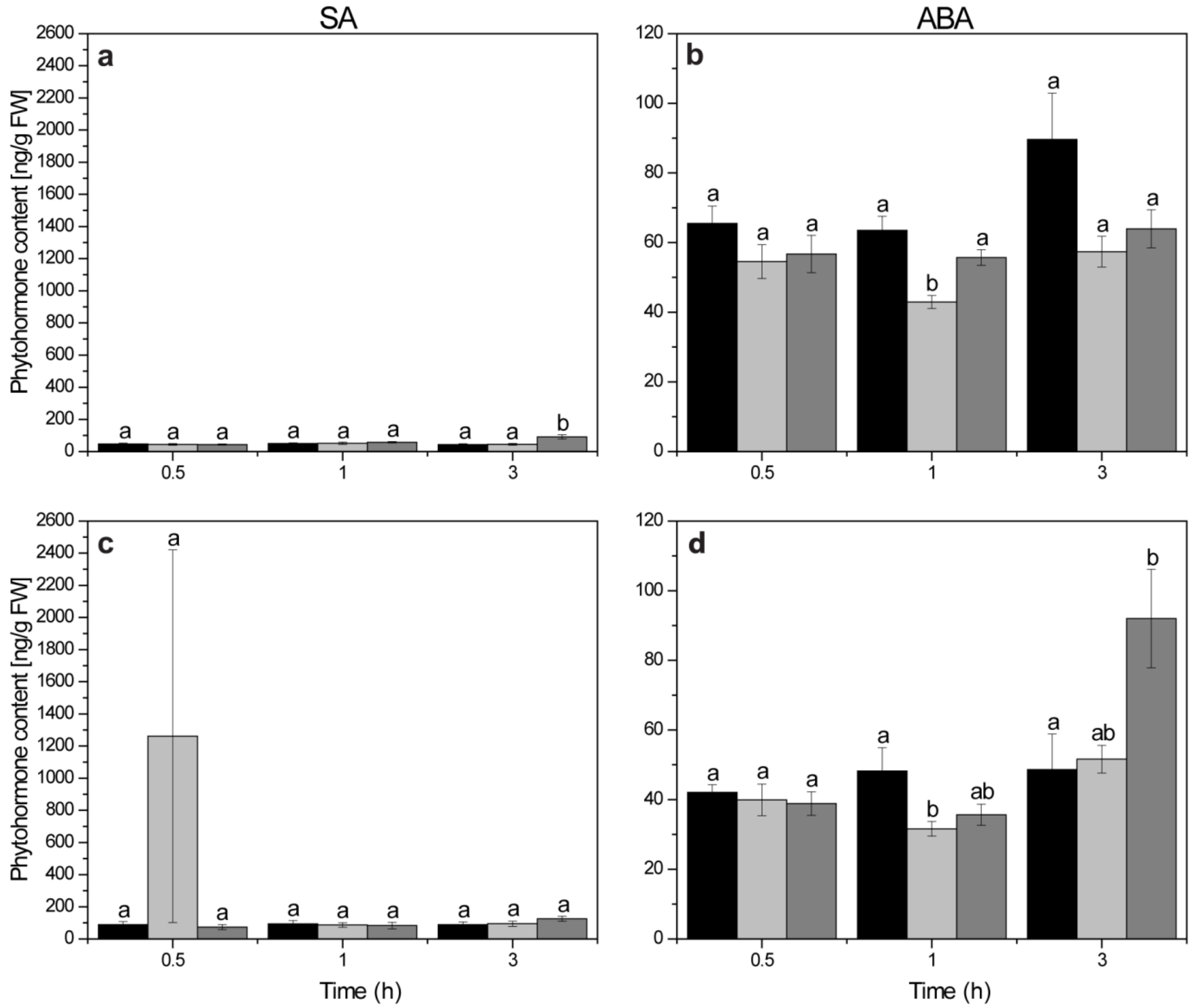

Supplementary Figure S3. Stable amount of salicylic acid and abscisic acid after wounding in *Ipomoea batatas* TN57.

(a-d) Salicylic acid (SA) and abscisic acid (ABA) levels after mechanical wounding by MecWorm (a, b; n = 10-11) and insect feeding by *S. littoralis* (c, d; n = 7-12) measured in *I. batatas* TN57 after 0.5 h, 1 h, and 3 h of treatment. Bars show the mean ± SEM of SA (a, c) and ABA (b, d) concentrations. Phytohormone levels were measured in locally wounded leaves (dark gray bars) and the adjacent unwounded systemic leaf (light gray bars). Leaves from undamaged plants were used as controls (black bars). Statistically significant differences between each treatment group after treatment were analyzed for each time point separately using one–way ANOVA or Kruskal-Wallis one-way ANOVA on ranks. Different letters indicate significant differences among groups for $p<0.05$, determined by Tukey's test.

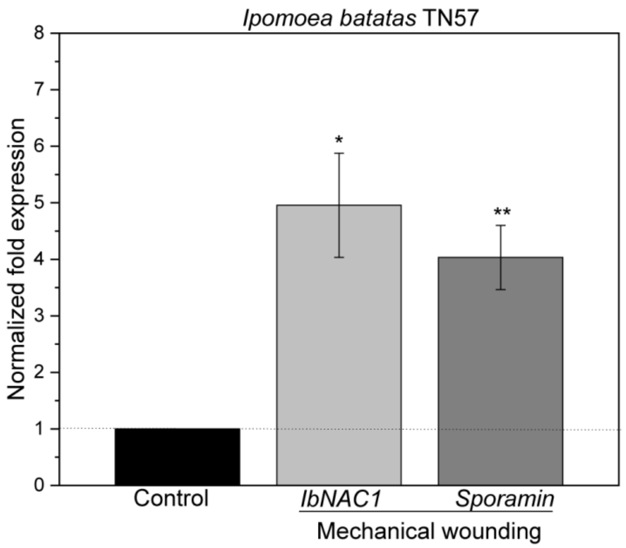

Supplementary Figure S4. Volatiles emitted by mechanically wounded plants induce *IbNAC1* and *Sporamin* in neighboring TN57 plants.

qRT-PCR of *IbNAC1* and *Sporamin* in unwounded TN57 leaves after incubation for 24 h with mechanically damaged neighboring plants. Bars represent the mean ± SEM of normalized fold *IbNAC1* (light gray bar) and *Sporamin* (dark gray bar) expression levels with undamaged plants (black bar) used as a respective control. Significance levels are indicated by the asterisks (* = $p<0.05$; ** = $p<0.01$) with n = 3. p (Cx*IbNAC1*) = 0.016; p (Cx*Sporamin*) = 0.005. Statistical analyses were performed for the treatment and the respective control with a Shapiro–Wilk normality test and subsequent one–sample t–test.

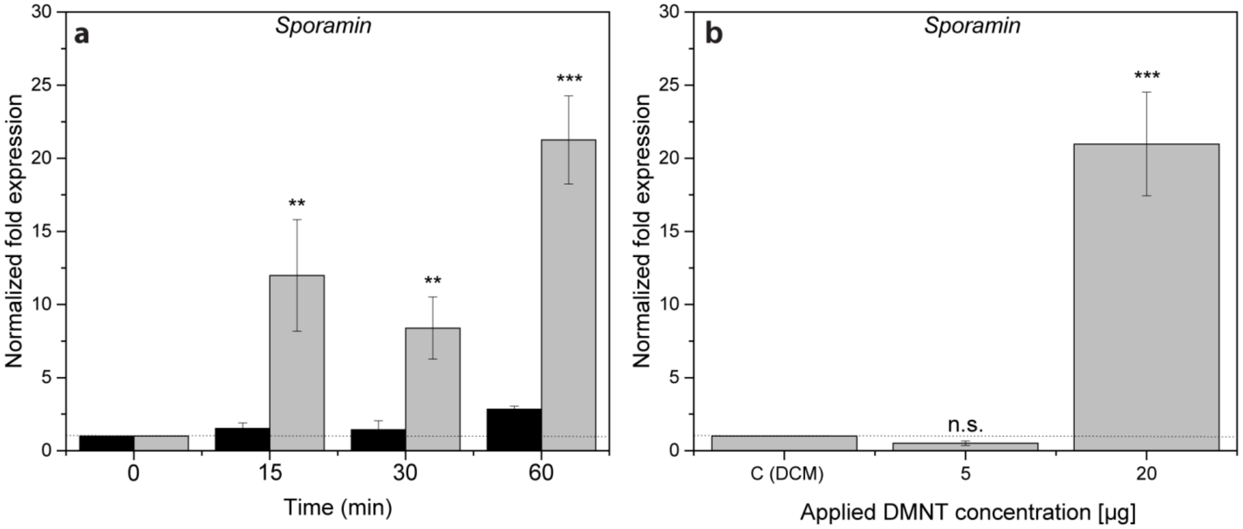

Supplementary Figure S5. Systemic induction of defense-related *Sporamin* by airborne DMNT occurs in a concentration- and time- dependent manner.

(a) Sweet potato plants (n = 3) incubated in an odorless glass container with 20 µg DMNT in a volume of 34 L (3.9 nM) dissolved in dichloromethane (DCM; light gray bars) for 15, 30 and 60 min. Plants incubated with DCM were used as control (black bars). (b) Sweet potato plants (n = 3) incubated for 1 h with either 5 µg or 20 µg of DMNT dissolved in pure DCM. Changes in the transcript level of Sporamin were determined in the 3rd fully expanded leaf of each plant. The gene fold expression change was calculated relative to DCM–treated control plants. Bars represent the mean ± SEM of normalized fold expression levels of *Sporamin*. Significance levels are indicated by the asterisks (n.s. = non-significant; ** $p<0.01$; *** $p<0.001$). (a) p (Cx*Sporamin* 15 min) = 0.009; p (Cx*Sporamin* 30 min) = 0.006; p (Cx*Sporamin* 60 min) < 0.001. (b) p (Cx*Sporamin* 5 µg) = 0.094; p (Cx*Sporamin* 20 µg) < 0.001 . Statistical analyses were performed for each treatment and the respective control with a Shapiro–Wilk normality test and subsequent one–sample t–test.

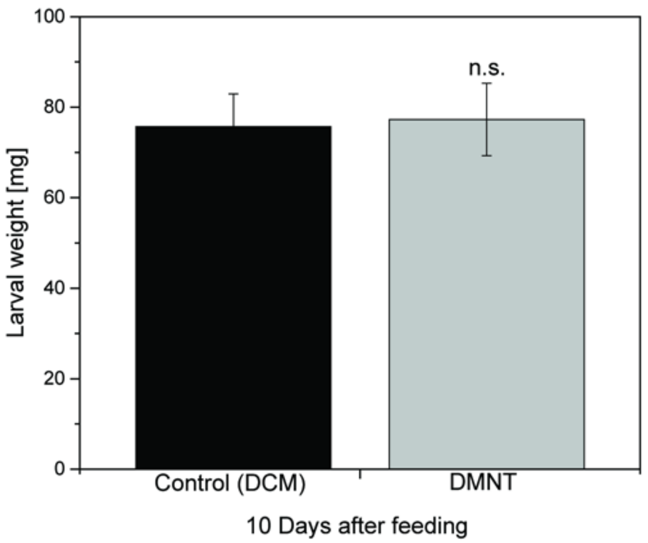

Supplementary Figure S6. DMNT is not toxic to *Spodoptera litura*.

Second instar *S. litura* larvae (n = 7) reared on artificial diet were exposed to DMNT (20 µl of 1 mg ml-1 DMNT in DCM) or 20 µl pure DCM (control) without direct physical contact in a glass container (34 L) at 25 °C. The larval weights were determined after 10 d of feeding. Bars represent the mean ± SEM of measured larval weight. Significance levels are indicated by n.s. = non-significant; p (CxDMNT) = 0.886. Statistical analyses were performed for the treatment and the respective control with a Shapiro–Wilk normality test and a subsequent t–test.

Supplementary Table S1. Identification of volatiles collected in the headspace of *I. batatas* TN57 and TN66 after mechanical wounding and feeding by *S. littoralis.*

See separate Excel file Supplementary Table S1

Supplementary Table S2. Quantification of volatiles (ng g^{-1} fresh weight) collected in the headspace of *I. batatas* TN57 and TN66 after mechanical wounding and feeding by *S. littoralis* relative to the internal standard.

See separate Excel file Supplementary Table S2

Supplementary Table S3. Primers used for real-time qPCR.

Primer name	Sequence
IbACTIN-2 F	GACTACCATGTTCCCCGGTA
IbACTIN-2 R	TTGTATGCCACGAGCATCTT
Sporamin F	TACTACATGTCTCCGCCATATGGG
Sporamin R	CTCAATCTTGAACTGGTTGCTATTGTC
IbNAC1 F	CGGCCGGGGATACAAATTTGTAAGCTT
IbNAC1 R	GAATCGGAATCCCGGCGGCATCTC
